# RNAfitme: a webserver for modeling nucleobase and nucleoside residue conformation in fixed-backbone RNA structures

**DOI:** 10.1186/s12859-018-2317-9

**Published:** 2018-08-22

**Authors:** Maciej Antczak, Tomasz Zok, Maciej Osowiecki, Mariusz Popenda, Ryszard W. Adamiak, Marta Szachniuk

**Affiliations:** 1 Institute of Computing Science & European Centre for Bioinformatics and Genomics, Poznan University of Technology, Piotrowo 2, 60-965 Poznan, Poland; 2 Poznan Supercomputing and Networking Center, Jana Pawla II 10, 61-139 Poznan, Poland; 3 Department of Biology, Adam Mickiewicz University, Umultowska 89, 61-614 Poznan, Poland; 4 Institute of Bioorganic Chemistry, Polish Academy of Sciences, Noskowskiego 12/14, 61-704 Poznan, Poland

**Keywords:** RNA 3D structure, Nucleoside modeling, 3D structure refinement

## Abstract

**Background:**

Computational RNA 3D structure prediction and modeling are rising as complementary approaches to high-resolution experimental techniques for structure determination. They often apply to substitute or complement them. Recently, researchers’ interests have directed towards in silico methods to fit, remodel and refine RNA tertiary structure models. Their power lies in a problem-specific exploration of RNA conformational space and efficient optimization procedures. The aim is to improve the accuracy of models obtained either computationally or experimentally.

**Results:**

Here, we present RNAfitme, a versatile webserver tool for remodeling of nucleobase- and nucleoside residue conformations in the fixed-backbone RNA 3D structures. Our approach makes use of dedicated libraries that define RNA conformational space. They have been built upon torsional angle characteristics of PDB-deposited RNA structures. RNAfitme can be applied to reconstruct full-atom model of RNA from its backbone; remodel user-selected nucleobase/nucleoside residues in a given RNA structure; predict RNA 3D structure based on the sequence and the template of a homologous molecule of the same size; refine RNA 3D model by reducing steric clashes indicated during structure quality assessment. RNAfitme is a publicly available tool with an intuitive interface. It is freely accessible at http://rnafitme.cs.put.poznan.pl/

**Conclusions:**

RNAfitme has been applied in various RNA 3D remodeling scenarios for several types of input data. Computational experiments proved its efficiency, accuracy, and usefulness in the processing of RNAs of any size. Fidelity of RNAfitme predictions has been thoroughly tested for RNA 3D structures determined experimentally and modeled in silico.

## Background

RNAs play a central role in all aspects of cell functioning. They constitute genomes of viruses and retroviruses. They have also been recognized as new targets in RNA diagnostics, molecular therapy, and nanotechnology. Biological functions and physicochemical properties of RNAs depend on their 3D structure and dynamics. The details of RNA structures have been mostly uncovered using high-resolution experiments of X-ray crystallography, NMR or electron cryo-microscopy. However, recent decades have resulted in the development of computational methods that substitute or complement experimental techniques and facilitate processing of their output data. Among them are the tools for RNA 3D structure prediction and modeling which have been applied in such fields as molecular and structural biology, RNA therapeutics or engineering of biomaterials [1].

Generally, predictive algorithms have located in one of two groups: (i) physics-based methods which proceed with de novo prediction, using dynamics simulation of a full-atom or coarse-grained RNA models; (ii) knowledge-based techniques that apply various structure templates in homology modeling. Until today, several methods have been released and acknowledged pretty reliable under these two groups [2–9]. Since 2010, their fidelity and applicability have been verified within RNA-Puzzles – a periodical, collective experiment for a blind RNA three-dimensional structure prediction [10–12]. Simulated RNA 3D models are submitted to RNA-Puzzles and evaluated using different measures, based on their comparison to target structures [13–17]. Successive puzzles reveal strengths and limitations of proposed algorithms and show what 3D structure elements or parameters cause the most significant problems. A lot of these problems can be solved by subsequent remodeling of the 3D structure [18, 19] or its repairing with [20] or without additional experimental data.

RNA structure modeling usually relies on computer-aided manipulation of the 3D models determined experimentally or predicted computationally. To varying degrees, it requires the user intervention that consists of different operations like removal, insertion, replacement, rotation or shifting of the tertiary structure fragments. Total or partial automation of the modeling process can be possible thanks to the use of problem-tailored optimization procedures, well-defined conformational constraints and structure data repositories. The latter ones constitute the search space for combinatorial algorithms which look for optimal conformation of analyzed RNA.

In contrast to proteins, the extent of possible RNA conformations is much complex. The RNA backbone is defined by six degrees of freedom. Thus, exploring RNA conformational space and constructing its components is a non-trivial task. An initial solution to this problem has proposed to build conformer libraries founded on torsion angles. Typically, α, β, γ, δ, ε, and ζ dihedral angles of sugar-phosphate backbone specify individual nucleotide unit of RNA [21]. An alternative approach is based on suites spanning two sugars and the intervening phosphate [22]. Each RNA suite is characterized by a combination of δ_i_, ε _i_, ζ _i_, α _i + 1_, β_i + 1_, γ _i + 1_, δ _i + 1_ angles, where *i*, *i + 1* denote the *i*-th and the (*i + 1*)-th nucleotide in the RNA chain. This concept has been applied to construct the library of RNA backbone conformers which encompassed nearly 50 discrete suites essentially free of steric clashes [23]. Modeling of large RNA 3D structures with the use of such dataset has appeared challenging [24]. However, backbone rotamer library has been employed for example in RCrane. This semi-automated system allows building full-atom RNA structure models by placing phosphates and bases in the electron density map [25]. The concept of backbone-dependent modeling has also been employed in RNA-Redesign [26], although this method is rather oriented on the design of new RNAs. Its primary goal is to find and optimize sequence variants of an RNA molecule that matches a fixed conformation of the sugar-phosphate backbone.

In our previous work [27], we have proposed a different approach by developing libraries of fixed-backbone-dependent RNA conformers. Members of these libraries have been selected as representatives of RNA conformational space that includes nucleoside residues collected from PDB-deposited RNA structures. Every conformer has been described by a set of torsional angles, atom coordinates, and covalent bond lengths. It constitutes particular structural template that can be used to model the global minimum energy conformation of nucleobase and nucleoside onto fixed coordinates of RNA sugar-phosphate backbone. Such concept has corresponded to that applied in protein structure homology modeling where prediction of amino acid sidechains has been based on the library of backbone-depended side chain rotamers [28, 29]. The same methodology has been followed in some webserver tools, e.g., SCWRL [30] and OPUS-Rota [31].

Here, we present RNAfitme, a versatile webserver tool for modeling of nucleobase and nucleoside residue conformations in fixed-backbone RNA 3D structures. It allows modeling, reconstructing, and remodeling from one to all residues in the RNA chain. RNAfitme method has adapted a graph-based algorithm to look for the optimal mutual arrangement of nucleobase/nucleoside residues in the RNA structure. The most important criterion followed by the algorithm is the minimization of steric clashes between predicted and fixed structure fragments. Several libraries [27] applied in RNAfitme define the space of fixed-backbone-dependent RNA conformers.

The webserver has appeared particularly well suited to the following applications. First is a reconstruction of full-atom RNA 3D model from the fixed-backbone atom coordinates. It is accompanied by the restoration of RNA secondary structure. In this application, RNAfitme allows studying what 3D structures can be reconstructed on a given sugar-phosphate backbone. Next, the tool can be applied to precisely remodel – in a given RNA structure – selected nucleobase/nucleoside residues pointed by the user. The latter application allows observing the influence of local modifications on the secondary and the tertiary structure of analyzed RNA. The third aspect concerns template-based prediction of RNA 3D model with a homologous RNA sequence(s) of the same size. This additional capacity of RNAfitme is restricted to cases which can be solved using substitutions only (insertions and deletions are not supported). Finally, let us add that in any usage scenario, the tool optimizes RNA 3D structure model by reducing steric clashes.

RNAfitme is free and open to all users without any login requirement. The webserver is publicly available at http://rnafitme.cs.put.poznan.pl/.

## Implementation

RNAfitme aims to model – in a fully-automated way – nucleobase and nucleoside residues in RNA 3D structure with preserving fixed coordinates of sugar-phosphate backbone atoms. The system kernel is a collection of conformer libraries and a graph-based optimization algorithm that builds the preliminary RNA 3D model. The algorithm’s tasks include: searching the library for candidate conformers; conformers’ reconstruction on the fixed-backbone; selecting the subset of promising candidates; controlling the reconstruction accuracy from a global perspective. The libraries create a discrete conformational space explored by the candidate search procedure. Additionally, RNAfitme includes a pre-processing module that validates and cleans an input data, and a post-processing one to execute energy minimization thus, improving the geometry of the preliminary 3D structure. In the latter step, the minimization encompasses only these structure fragments that were remodeled in RNAfitme. It is conducted using CHARMM force field by NAMD method [32]. Next sections provide the details of the RNAfitme system components.

### Input data and its pre-processing

At the input, the user should upload PDB file that includes either full-atom RNA 3D structure or sugar-phosphate backbone atoms only, or give a PDB identifier of the structure to be remodeled. In the latter case, the PDB file is automatically downloaded from the Protein Data Bank [33]. Depending on the usage scenario, a sequence in FASTA format can be also entered. It is required if the user aims to predict a homologous structure or remodel selected nucleobase/nucleoside residues. The input sequence is case-sensitive. All residues denoted by lower case letters are treated as fixed. All upper case letters show the ones which are to be reconstructed/remodeled. At the input, the user also selects a processing mode (nucleobase or nucleoside residue modeling) and a conformer library. Finally, a decision on energy minimization application can be made (be default this option is selected). If the user enters an email address, the input data and link to the result page will be emailed when ready. Right after clicking the “Run” button, a pre-processing of the input data begins.

First, the input 3D structure is cleared of atoms that are not compatible with the PDB format. Next, a procedure checks if the required atoms are present in the input data. In the nucleoside mode the input 3D structure should include backbone atoms (O5’,C5’,C4’,C3’,O3’). In the nucleobase mode – backbone and sugar ring atoms (O5’,C5’,C4’,C3’,O3’, O4’,C1’,C2’,O2’). All the remaining atoms of remodeled/reconstructed fragments are removed from the input structure. The pre-processing module also verifies if the sequence length (if given) and input RNA chain length are equal. The lower bound for the input data size has been set to 3 nucleotide residues. No upper bound is defined. Finally, from input coordinates, the system computes lengths of covalent bonds, values of torsional angles along the backbone, and pseudotorsion angles. This data is used by optimization algorithm in further processing.

### Conformer libraries

RNAfitme has a built-in repository which consists of five conformer libraries. These are: (i) Neural gas clustering - Euclidean distance - High-resolution RNA structures (NG-E-HR); (ii) Neural gas clustering - MCQ distance - High-resolution RNA structures (NG-M-HR); (iii) Neural gas clustering - Euclidean distance - 23S rRNA (NG-E-23S); (iv) K-medoids clustering - Euclidean distance - all RNA structures (KM-E-ALL); (v) Neural gas clustering - Euclidean distance - all RNA structures (NG-E-ALL). Their construction has been described in detail in [27]. By default, the first one (NG-E-HR) is used in the computational workflow. However, any of them can be applied upon the user selection. The libraries constitute conformational space which is explored when nucleobase/nucleoside residues are searched for to be reconstructed in the RNA structure.

In general, each library was generated following the same protocol [27]. In every case, the building process started with a selection of RNA 3D structures being a subsequent data source. For NG-E-HR and NG-M-HR, 553 high resolution (≤ 2.4 Å) RNA structures were collected (*HR* set). In the case of NG-E-23S, the crystal structure of the *Haloarcula marismortui* ribosomal subunit (PDB ID: 3CC2) [34] was chosen (*23S* set). For KM-E-ALL and NG-E-ALL, we have taken all RNA 3D structures (*ALL* set) deposited in the Protein Data Bank [33] at that time (i.e., 2608 structures from RNAs and RNA complexes). All complete, unmodified nucleotide residues were extracted from selected source 3D structures and annotated (i.a., a vector of torsion angle values was computed for each considered residue). Thus, the *HR* set included 65,134 residues, the *23S* set – 2876, and the *ALL* set – 1,743,940. Next, the residues in every set were hierarchically clustered using either neural gas (NG) [35] or k-medoids (KM) [36] algorithm, and one of two distance measures, Euclidean (E) or MCQ (M) [16]. Finally, each cluster representative (prototype) was identified and stored in the appropriate library. This way, we have obtained 12 different libraries which have been validated and evaluated in dedicated computational experiments [27]. Five libraries providing the most variable and accurate selection of conformers were selected for RNAfitme.

Every library has a different size and hierarchical structure. NG-E-HR contains 282 groups resulting from 1st-stage clustering (based on α, β, γ, δ, ε, ζ angles). They include 1742 elements resulting from 2nd-stage clustering (based on the χ angle). For the remaining libraries, these numbers are as follows: 58/319 in NG-M-HR, 231/580 in NG-E-23S, 16/32 in KM-E-ALL, and 381/1637 in NG-E-ALL. Detailed information about residue types representation in each library is included in [27]. Each conformer is described by a complete set of torsional angles (α, β, γ, δ, ε, ζ, τ_0_, τ_1_, τ_2_, τ_3_, τ_4_, χ), coordinates of all atoms, and lengths of covalent bonds.

### Optimization algorithm

The RNAfitme method has been inspired by the approach proposed for proteins [28]. Our optimization algorithm for nucleobase/nucleoside modeling follows the same concept, taking into account more complex properties of RNA molecules. The algorithm builds a preliminary RNA 3D model, minimizing its repulsive steric energy. An application of such optimization criterion aims to reduce the number of invalid contacts in the modeled structure. The algorithm operates on the library of conformers treated as the initial search space and reduces it to finally identify single conformer per each analyzed nucleobase/nucleoside residue. The method proceeds according to a multi-stage routine. However, if the solution (i.e., an optimum set of conformers) is found earlier, i.e., after some intermediate step, succeeding ones are discarded.

#### Step 1: Finding candidate conformers

For every reconstructed/remodeled residue in the nucleoside mode, the most similar ribose ring conformers are looked for in the selected library. Their adjustment to the fixed-backbone is assessed based on MCQ score [16] computed upon corresponding backbone torsion angles. For every residue, a set of candidate conformers is identified. All the candidates found in this step build the conformational search space, while the others are discarded. Note that the numbers of candidate conformers differ between the residues.

#### Step 2: Selecting promising candidates

To optimize further computation, the number of candidates is reduced to a maximum of ten for every residue. In each case, the nucleobase/nucleoside residue is reconstructed on the fixed-backbone based on the torsional angle-driven characteristics of a conformer. Next, RMSD (Root Mean Square Deviation) [37] score is computed between the reconstructed residue and its counterpart from the candidate set. Up to ten conformers with the lowest RMSD value are selected as most promising candidates.

#### Step 3: Computing interatomic energies

Two types of energy terms are calculated for every promising candidate *c*_*m*_ that remained in the conformational search space. First, the energy *E*_*fixed*_(*c*_*m*_) between *c*_*m*_ and the fixed part of the input structure is computed. Second, for each yet unresolved conformer *c*_*n*_, the method estimates interatomic energy *E*_*pair*_(*c*_*m*_, *c*_*n*_). In all the cases, the energy is approximated using Lennard-Jones potential [38].

#### Step 4: Eliminating energetically unfavorable conformers

In this step, the dead-end elimination (DDE) procedure [30] is performed to remove conformers which cannot be part of the global minimum energy conformation. Our version of the method uses Goldstein criterion [30]. It states that a given conformer *c*_*m*_(*r*_*i*_) found for residue *r*_*i*_ can be pruned from the search space if there is another conformer *c*_*n*_(*r*_*i*_) such that it always contributes lower energy with all other neighboring conformers present in the search space than *c*_*m*_(*r*_*i*_). DDE is applicable in cases when more than one candidate conformer is considered for a single nucleobase/nucleoside residue.

#### Step 5: Creating residue-residue interaction network

At this stage, only these residues are considered for which more than one conformer is present in the conformational search space. Such residues are taken to create a residue-residue interaction network which has a form of an undirected graph *G*. Every residue *r*_*i*_ from the considered set corresponds to a node *v*_*i*_ in graph *G*. Two nodes *v*_*i*_, *v*_*j*_∈*G* are connected with an edge if there exists at least one pair of conformers *c*_*m*_(*r*_*i*_), *c*_*n*_(*r*_*j*_) for which a pairwise interaction energy is positive: *E*_*pair*_(*c*_*m*_(*r*_*i*_), *c*_*n*_(*r*_*j*_)) > 0.

#### Step 6: Identifying connected components

In this step, a procedure runs to find all connected components in the residue-residue interaction network. *K* will denote the number of identified connected components. A connected component in an undirected graph *G* is defined as a subgraph *G’*⊂*G* in which there is a path between any two nodes of *G’* and which is not connected to any other node that does not belong to *G’*.

#### Step 7: Decomposing connected components

A depth-first search algorithm is performed on every connected component *G*_*k*_*’* of *G* (*k* = 1..*K*). It decomposes *G*_*k*_*’* into a block-cut tree (i.e., a tree of biconnected components, where every biconnected component is a set of residues located close to one another and far from other residues). Next, articulation points (i.e., nodes shared by different biconnected components) are identified in every subgraph *G*_*k*_*’*.

#### Step 8: Selecting best conformers

At this stage, we find the minimum energy for each connected component. It is done while a branch-and-bound backtracking algorithm performs to solve every block-cut tree. As a result, each articulation point is associated with a subset of conformers selected for the corresponding residues and their total interatomic energy. Articulation points are then recursively collapsed, and their energy propagates further. The process stops when no articulation point exists.

### Preliminary model post-processing

The final stage of processing in the RNAfitme system is to minimize the repulsive steric energy of the preliminary 3D model. This step may be omitted if the user unchecks the “Minimize 3D model energy” option. Although the final minimization is not mandatory, its application is suggested. Therefore, the option is enabled by default.

The energy minimization procedure is conducted by NAMD, using CHARMM force field [32]. NAMD improves the preliminary RNA model geometry by smoothing the structure and further reducing steric clashes. The minimization addresses only these atoms of the 3D structure that were reconstructed/remodeled by RNAfitme in the previous steps. It is possible thanks to the adapted NAMD configuration and own protocols prepared for the needs of the RNAfitme system.

### Output data

At the output, the user obtains an RNA three-dimensional structure model in the PDB file format. The model is visualized using JSmol which has been incorporated into the RNAfitme system. In the visualization, fixed atoms are dark blue, while the remodeled ones are green. Visualization panel allows for interactive manipulation. The resulted 3D model can be downloaded and saved in various file formats (GIF, JPG, PNG, PNG + JMOL, POV-Ray). A popup menu to allow such operations shows up after clicking the right mouse button in the panel area. The log file is provided along with the output 3D model. It enumerates all computing steps performed by RNAfitme and shows the result of every intermediate step. Computing time is also given.

All the presented output data can be downloaded in a zipped archive by selecting the checkbox and clicking “Download selected results”. The result page also allows to run RNAfitme once again for the same RNA 3D structure with input parameters changed. This is possible after clicking “Run with different parameters” button and selecting the other set of input parameters.

## Results and discussion

In this section, we present and discuss the results of RNAfitme experimental runs in several usage scenarios and for selected examples of RNA structures. We analyze the results given by the webserver in the case of structure processing in nucleobase and nucleoside modeling mode, and we confirm the accuracy of obtained 3D models under global quality assessment.

**Fig. 1:**
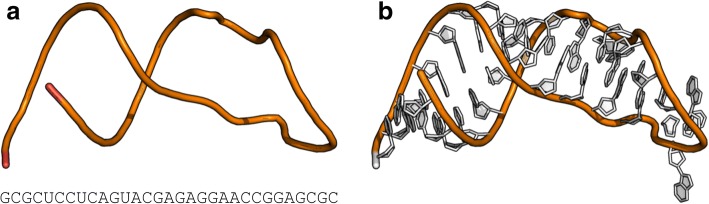
**a** Example input data and (**b**) full-atom RNA 3D structure reconstructed in the nucleoside residue modeling mode of RNAfitme. Both views were generated in PyMOL [43]

In the first computational experiment, we run RNAfitme to reconstruct full-atom 3D structure based on the fixed-backbone and RNA sequence provided by the user. For this experiment, we selected crystal structure of a 31-mer SRD RNA inhibitor bound to the ribotoxin restrictocin from *Aspergillus restrictus* (PDB ID: 1JBR, chain D) as a reference [39]. We prepared the input data for RNAfitme by removing from the PDB file all coordinates except these of atoms from the sugar-phosphate backbone. Then, we uploaded the data, i.e., the altered PDB file and the following original sequence: GCGCUCCUCAGUACGAGAGGAACCGGAGCGC (Fig. 1a). RNAfitme was run in the nucleoside residue modeling mode operating on the NH-E-HR library with structure minimization option. As a result, we obtained a full-atom 3D structure as presented in Fig. 1b. To evaluate the accuracy of modeling, we assessed the reconstructed full-atom 3D model in the context of the reference structure. The RMSD computed based on a set of reconstructed atoms was 1.349 Å. Following the assumptions, RNAfitme did not violate coordinates of atoms in the sugar-phosphate backbone. Next, we run commonly used MolProbity tool [14] to check if steric clashes occurred in the 3D model generated by RNAfitme, and we obtained Clash score = 0.99 (99th percentile). For comparison, it should be mentioned that the Clash score for the reference structure was 2.99 (98th percentile). Thus, RNAfitme not only reconstructed the 3D structure but also reduced steric clashes found in the experimental model. To complete the evaluation we compared the model generated by RNAfitme and the reference one on the secondary structure level. With using RNApdbee [40, 41] run with the default input parameters, we annotated the secondary structures of both to learn that they were identical (their INF score = 1.0). It proved that RNAfitme correctly restored all secondary structure interactions.

**Fig. 2:**
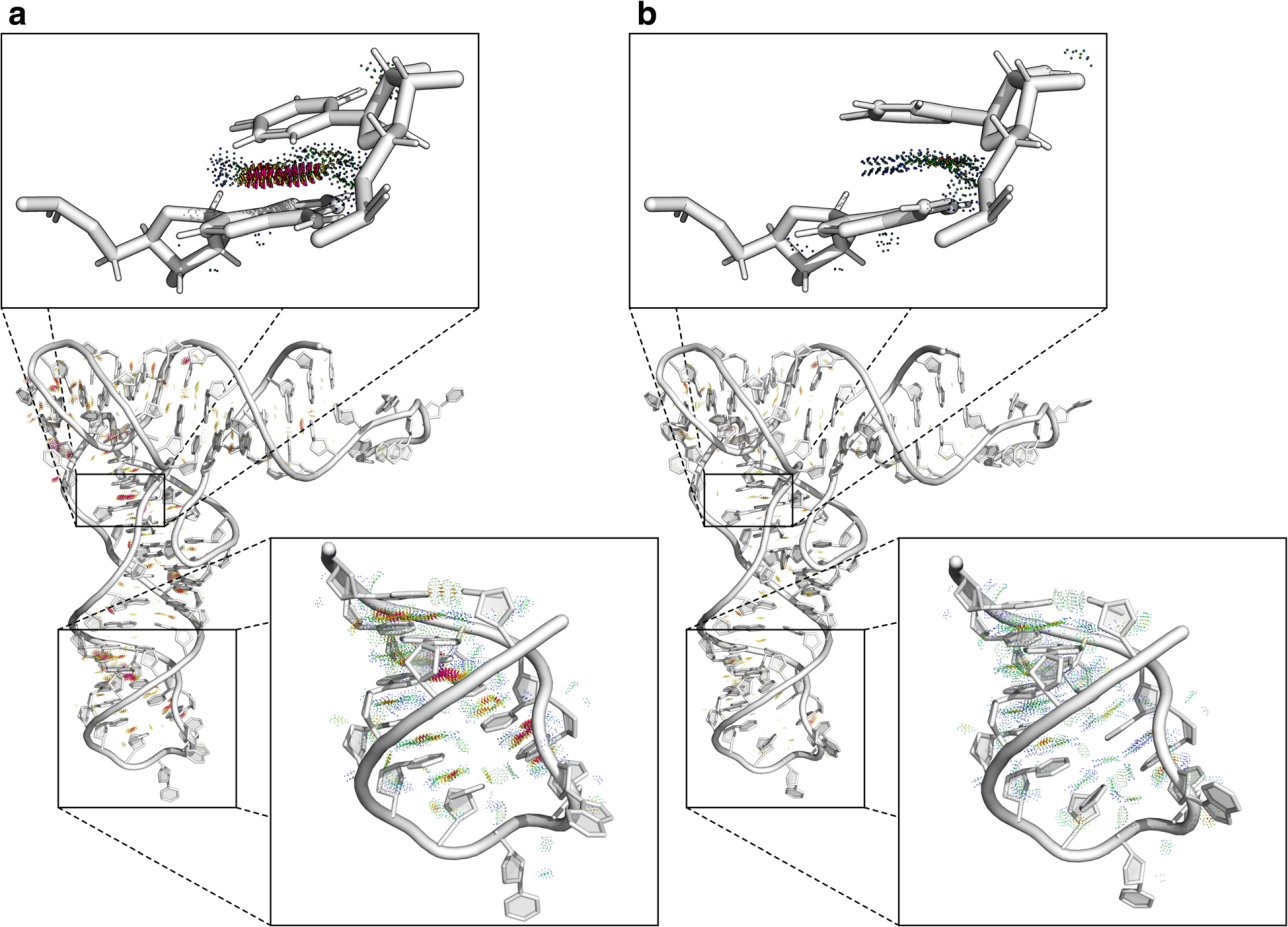
The 3D structure of glutamine tRNA bound to the cognate synthetase (1EXD, chain B) (**a**) before and (**b**) after processing by RNAfitme in the nucleobase remodeling mode. Zoomed-in views present A921 and U948 residues, and C928-G942 segment with clashed atoms in red

The second experiment aimed to remodel the 3D structure to correct it by reducing steric clashes. We selected glutamine tRNA bound to the cognate synthetase (PDB ID: 1EXD, chain B) [42] as an example. We processed this 73 nt-long RNA 3D structure with MolProbity [14] to find that its Clash score was equal to 38.95 (9th percentile). Thus, we uploaded this 3D structure to the RNAfitme input, and we run the server in the nucleobase remodeling mode with the use of NG-E-HR library and energy minimization option. In this experiment, RNAfitme algorithm proceeded remodeling of all nucleobases present in the input 3D structure while keeping the sequence and the secondary structure unchanged. The Clash score computed for the output model was 11.5 (65th percentile). Detailing, the input structure contained 90 incorrect contacts which involved 143 atoms, while in the output model, there were only 27 of these contacts left unchanged, in which 40 atoms participated. Let us recall that due to the adopted rule of keeping all atoms of sugar-phosphate backbone fixed, RNAfitme was not able to remove steric clashes of these atoms. Moreover, in the nucleobase remodeling mode, only nucleobase atoms are not fixed. Thus, only clashes that involved these atoms could be removed. One of the most clashed fragment of the structure encompassed residues labeled as A921 and U948 (original numbering from the PDB file). Over 30% of atoms in these residues collided creating invalid contacts. RNAfitme was able to remove all clashes in this area. Other parameters computed by MolProbity were also improved: probably wrong sugar puckers were reduced from 16 to 0%, and 50% of bad angles were repaired. To check to what extent the structure changed, we calculated RMSD score of the remodeled versus the input structure. RMSD over nucleobase atoms reached the value of 0.784 Å. We also checked if torsion angles had changed. It appeared that two χ angles in the output model had different orientation (syn/anti) than their counterparts in the input structure. MCQ computed for the set of χ angles was equal to 11.12 degrees. Figure 2 displays the visualization of RNAfitme input (Fig. 2a) and output (Fig. 2b) structures of the analyzed molecule. The views of A921-U948 residues and C928-G942 segment are zoomed-in to show this inaccurate part better. Clashed fragments in the structure have been colored red. Steric clashes between atoms of selected residues are visualized in PyMOL using PyMOLProbity plugin [43].

**Fig. 3:**
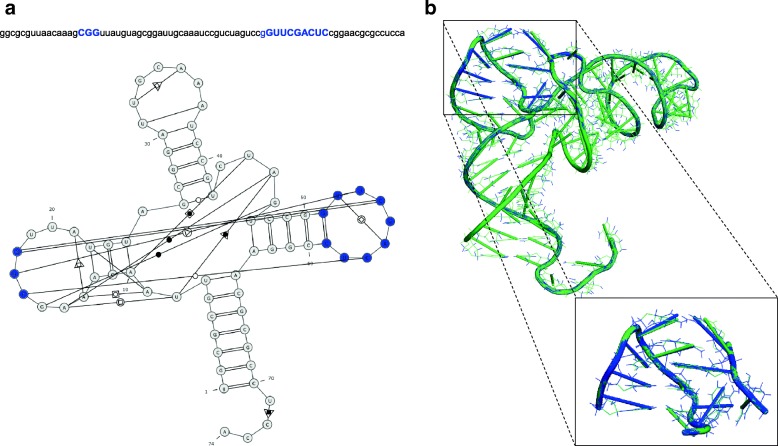
**a** The sequence and secondary structure of cysteine tRNA bound to the cognate synthetase (PDB ID: 1U0B, chain A), and (**b**) its output 3D structure after processing by RNAfitme. Remodeled nucleoside residues are colored blue

In the third single-case experiment we run RNAfitme to remodel user-selected nucleoside residues in the input RNA 3D structure. We chose the crystal structure of cysteine tRNA bound to the cognate synthetase (PDB ID: 1U0B, chain A) [44] as example input. Initial validation of this RNA 3D structure quality was made using MolProbity [14]. It showed Clash score equal to 13.89 (54th percentile) with 33 invalid interatomic contacts resulting from steric clashes occurrence. 42% of them (namely, 14 contacts) appeared between residue pairs of which at least one was between G51 and C59 residue (according to PDB numbering of residues, the range is G53–C61). A study of the secondary structure of this molecule (cf. Fig. 3a) revealed that these residues belonged to the apical loop which interacted with the other loop where four clashed contacts were observed between C16 and G18 residues (numbers of these residues in the PDB file are different: C16, G18, G19). Thus, we decided to remodel the considered 3D structure focusing on these 12 problematic residues (colored blue in the sequence and the secondary structure diagram in Fig. 3a). We uploaded the PDB file and the given tRNA sequence at the RNAfitme input. The input sequence was encoded using lower- and uppercase letters. Lowercase ones denoted the fixed part of the input 3D structure, while the uppercase letters pointed the residues to be remodeled: ggcgcguuaacaaagCGGuuauguagcggauugcaaauccgucuaguccgGUUCGACUCcggaacgcgccucca. Next, RNAfitme was run in the nucleoside residue modeling mode operating on the NG-E-HR library with energy minimization option selected. MolProbity [14], used to examine the output 3D structure showed a substantial improvement in the structure’s quality. The Clash score computed for the entire structure after remodeling was 8.01 (82nd percentile). MolProbity enumerated 19 invalid interatomic contacts remained. Among them, 3 were still observed in the remodeled loops. It means that RNAfitme was able to remove 83% of steric clashes in the selected area of the input 3D structure. The remaining 17% resulted from sugar-phosphate backbone abnormalities that our method could not compensate. Next, we applied RNApdbee 2.0 [41] to verify if expected base pairs were reconstructed correctly in the resultant structure. It appeared that all base pair patterns were restored, although during the remodeling process one non-canonical pair, G18–C56, was changed into the canonical one. It caused the value of INF score computed for canonical interactions dropped from 1 to 0.976. However, global INF was equal to 1. Finally, we investigated the global shape of the molecule. RMSD score computed over remodeled residues, equal to 0.441 Å, showed that the structure changed to a small extent. The 3D structure obtained from RNAfitme is visualized in Fig. 3b. Remodeled nucleoside residues are colored blue. Zoomed-in view of the remodeled area gives a more clear picture of the introduced changes. Remodeled nucleoside residues (colored blue) are superimposed on their original counterparts (in green).

**Fig. 4:**
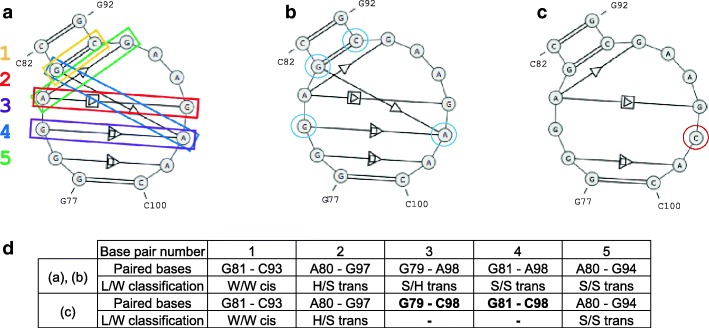
The secondary structure of kink-turn motif 23S KT-7 from *Haloarcula morismortui* ribosomal subunit (1S72) (**a**) before and after processing by RNAfitme (**b**) to remodel three base pairs, and (**c**) to introduce single point mutation; and (**d**) output models’ base pair details

The fourth experiment aimed to check how RNAfitme method performed for non-canonical base pairs, and mutations of base pairs introduced into the structure. As a test example, we selected the kink-turn motif 23S KT-7 cut out from *Haloarcula morismortui* ribosomal subunit (PDB ID: 1S72) [45]. This recurrent RNA motif sequence is gggagc-gcgaagaac. It has one canonical and four non-canonical base pairs. The secondary structure, annotated by RNApdbee [41], is displayed in Fig. 4a, where the considered base pairs have been enumerated. The motif was processed in several runs of RNAfitme. All of them were performed with NG-E-HR library and energy minimization on. In the first two, we applied the nucleobase and nucleoside residue remodeling mode for the following input sequence: ggGaGcgCgaagAac. Note that RNAfitme allows processing multichain structures, but sequences of individual chains should be concatenated at the input with no additional delimiters between them. Thus, four nucleobases/nucleoside residues (surrounded by blue circles in Fig. 4b) were remodeled, while the remaining ones were kept fixed. In both cases, all canonical and non-canonical base pairs were correctly rebuilt, and no clashes were introduced (cf. Fig. 4b, d). In the following two runs (nucleobase/nucleoside residue remodeling), we introduced a single point mutation by entering the following input sequence: gggagcgcgaagCac. It means that A98 from the original structure was exchanged for C. Let us notice that in the original structure, A98 was paired with G79 (S/H trans) and G81 (S/S trans). As expected, the introduced mutation caused a change in base pairing (cf. Fig. 4d). According to the isostericity matrix [46], S/S trans base pair G81–C98 could not be kept, while S/H trans G79–C98 could or could not retain. RNAfitme has broken both base pairs (Fig. 4c, d), what was revealed when we annotated the output 3D model by RNAView [47].

Next computational experiment aimed to examine RNAfitme performance and fidelity with the NG-E-HR, the default conformer library used in the system, and compare it to application of other libraries. For this experiment, we constructed a set *S19* of representative RNA 3D structures deposited in the Protein Data Bank [33]. The dataset contained 19 non-redundant, single-stranded RNA 3D structures with various sizes (37–229 nucleotide residues) and established secondary structure architecture (12–68 canonical base pairs). Every structure from *S19* was uploaded to RNAfitme input and processed in both, nucleobase and nucleoside remodeling mode, with NG-E-HR library and energy optimization option selected. Next, we compared the output 3D models with the original structures to find if the MolProbity-reported steric clashes were reduced. We also investigated the extent to which the tertiary and the secondary structure changed. In the first case, we analyzed RMSD of the output model with respect to the input (reference) structure. For the nucleobase remodeling mode, RMSD was computed over the following atoms: C2, C4, C5, C6, C8, N1, N2, N3, N4, N6, N7, N9, O2, O4, O6. For the nucleoside remodeling mode, the subset of considered atoms included: O4’, C1’, C2’, O2’, C3’, C4’, C2, C4, C5, C6, C8, N1, N2, N3, N4, N6, N7, N9, O2, O4, O6. To analyze the secondary structures, we used their extended dot-bracket representations that encoded canonical base pairs only. The secondary structures were annotated using RNApdbee 2.0 [41] with the default settings. Based on dot-bracket strings, we computed INF measure [13] to find whether the remodeling process influenced the secondary structure. As presented in Table 1, on the average clashes reduced by half in the nucleobase modeling mode, and by three-quarters in the nucleoside modeling mode. Such a relationship results from the fact that in the second case more substantial part of the 3D structure is subject to modifications. It can also be seen that in most cases RMSD does not exceed 1.0. Obviously, it is larger in the case of nucleoside mode. As for the secondary structure, we find over 90% of it is preserved in every case. For most structures, INF score is bigger in the nucleobase mode. This multi-case experiment proves that RNAfitme is a reliable tool for RNA 3D structure remodeling. It also shows that NG-E-HR library defines an efficient space for nucleobase and nucleoside conformation rearrangement within the given fixed 3D structure. We have also run RNAfitme with other conformer libraries to process structures from the *S19* dataset, and we analyzed the accuracy and quality of the resulting RNA 3D models. For every library, we computed average values of RMSD, INF and Clash score (Table 2). The average results do not differ significantly. However, for a single RNA structure, a choice of the library can influence the accuracy of the output model. Thus, we suggest the user tried an application of several libraries to find the optimum 3D structure that meets the expectations.

**Table 1:** The results of RNAfitme using NG-E-HR library to process RNA 3D structures from *S19* dataset

PDB ID: Chain	Chain length	Base pairs	Clash score	Nucleobase remodeling mode			Nucleoside residue remodeling mode		
				RMSD [Å]	INF	Clash score	RMSD [Å]	INF	Clash score
1CX0: B	72	22	15.12	0.468	1.000	9.50	1.019	1.000	8.65
1EXD: B	73	19	38.35	0.784	0.975	11.50	1.151	0.919	5.11
1FFY: T	75	22	19.77	0.813	1.000	15.31	0.761	1.000	6.21
1GID: A	158	48	41.62	0.636	1.000	14.55	1.035	0.936	7.28
1I6U: C	37	15	6.72	0.779	1.000	3.36	0.915	0.931	0.84
1MMS: C	58	14	8.05	0.897	0.966	5.89	0.804	0.966	3.22
1U0B: A	74	20	13.89	0.387	0.976	9.70	0.626	0.976	2.53
1UN6: E	61	17	16.26	0.917	0.946	8.12	0.979	0.915	3.55
1WZ2: C	88	24	23.98	0.622	0.958	13.40	1.085	0.914	8.82
1Y0Q: A	229	68	53.95	0.753	0.963	24.71	0.836	0.955	9.78
2J00: W	76	18	31.91	1.048	0.949	16.78	1.598	0.919	6.14
2PXL: B	47	14	7.24	0.462	1.000	3.29	0.743	0.964	1.31
3ADB: C	92	33	11.78	0.541	1.000	5.39	0.676	0.969	2.36
3AM1: B	81	30	33.28	0.529	1.000	15.30	0.835	0.966	8.80
3CUL: C	92	26	13.88	0.548	0.964	4.73	0.787	0.944	0.68
3IAB: R	46	12	8.78	1.237	1.000	5.40	1.410	1.000	2.03
3IQP: A	94	31	24.91	0.856	0.984	16.07	0.771	0.967	6.89
3IWN: A	93	28	24.97	0.800	0.982	16.63	1.174	0.906	11.64
3OFQ: B	117	34	51.89	0.920	0.985	21.46	1.083	0.955	8.74
Average value:			23.49	0.737	0.981	11.64	0.963	0.953	5.50

**Table 2:** Average values of accuracy (RMSD, INF) and quality (Clash score) measures for *S19* dataset processed by RNAfitme

Conformer library	Nucleobase remodeling mode			Nucleoside residue remodeling mode		
	RMSD [Å]	INF	Clash score	RMSD [Å]	INF	Clash score
NG-E-HR	0.737	0.981	11.64	0.963	0.953	5.50
NG-M-HR	0.744	0.979	12.63	1.475	0.901	7.39
NG-E-23S	0.751	0.990	13.20	1.111	0.951	6.07
KM-E-ALL	0.749	0.989	11.67	1.122	0.950	7.91
NG-E-ALL	0.703	0.978	12.75	1.065	0.975	4.13

## Conclusions

Experimental and predictive methods often produce RNA 3D structure models with poor quality [1, 48–50]. It imposes the refinement step to reach the right level of accuracy. The departure of the structure from an ideal stereochemistry, relatively small in the case of high-resolution X-ray RNA structures, can be unacceptable for predicted RNAs. Thus, specialized remodeling tools are required which has been revealed in succeeding RNA-Puzzles challenges during the quality assessment phase [10–12].

In this paper, we have addressed the problem of RNA 3D structure remodeling in the global and local perspective. We have anchored our solution on the concept of fixed sugar-phosphate backbone and libraries of nucleoside residue conformers. It has been implemented in RNAfitme webserver which primarily allows the user to study what RNA 3D structure can be built on the given sugar-phosphate backbone. RNAfitme can be applied in several usage scenarios. First, it enables reconstructing a full-atom model of RNA based on its sugar-phosphate backbone and the sequence. Second, it is suitable for precise remodeling of indicated RNA structure fragments with accuracy to a single nucleobase or nucleoside residue. Third, it can be used to predict homologous RNA 3D structure from the given sequence and three-dimensional template if the prediction can be done using substitutions only. It can be used to validate the results of 3D structure-based RNA design. Finally, it appears efficient in reducing inappropriate interatomic contacts involving nucleobase and nucleoside residue atoms and, thus, it can be useful in improving the RNA models submitted to RNA-Puzzles. This is because the optimization algorithm used in the modeling of preliminary RNA 3D structure, and the energy minimization NAMD protocol follow the criterion of minimizing steric clashes. However, it should be underlined that RNAfitme by design cannot repair backbone inaccuracies. Currently, one of the central assumptions of our tool is that all atoms included in the backbone are fixed. This fact, combined with the primary goal of clash reduction, in some cases may introduce unwanted artifacts. For example, substituting a pyrimidine with a purine in the canonical base pair can introduce planarity violation.

In the future, we plan to extend the functionality of RNAfitme by allowing to break the sugar-phosphate backbone inviolability. It will help in broadening the range of possible 3D structure modifications and introduce more flexibility into the remodeling process. Such a change can improve the accuracy of predicting the pseudoknotted RNA structures which currently constitute quite a significant problem of computational prediction methods [51]. Future plans include also implementation of procedures to support modified residues in the input RNA structure. Finally, we are going to relax the input validation function to allow conditional processing of RNA backbone with gaps.

## Availability and requirements

Project name: RNAfitme webserver.

Project home page: http://rnafitme.cs.put.poznan.pl

Operating system(s): Platform independent.

Programming language: Java 1.7.0, Spring.io.

Any restrictions to use by non-academics: no restrictions.

## Abbreviations

DDE: Dead-End Elimination
INF: Interaction Network Fidelity
MCQ: Mean of Circular Quantities
NMR: Nuclear Magnetic Resonance
PDB: Protein Data Bank
RMSD: Root Mean Square Deviation
